# Efficacy of Family Health Conversations on Mental Health, Family Wellbeing, and Family Functioning for Parents of Infants Requiring Mechanical Respiratory Support During Neonatal Intensive Care

**DOI:** 10.1177/10748407251357216

**Published:** 2025-08-03

**Authors:** Marie Åberg Petersson, Carina Persson, Johan Israelsson

**Affiliations:** 1 Region Kalmar County, Sweden; 2 Linnaeus University, Kalmar, Sweden; 3 Kalmar County Hospital, Sweden

**Keywords:** Family Health Conversation, neonatal intensive care, parents’ mental health, family wellbeing, family functioning

## Abstract

Having an infant requiring care in a neonatal intensive care unit (NICU) is challenging for parents. The aim was to investigate the effects of the Family Health Conversation (FamHC) model on self-reported mental health, family wellbeing, and family functioning in parents of infants requiring mechanical respiratory support during NICU care. This interventional study included 147 parents (72, intervention group; 75, control group). All participants received a study-specific questionnaire at three time points. The intervention trended toward positive effects on mental health, family wellbeing, and family functioning. However, all measurements showed considerable variation, and the estimated effects were not statistically significant at the 0.05 level. Regardless of the intervention, mental health symptoms decreased over time, whereas family wellbeing and functioning remained stable. To conclude, although the intervention trended favorable for all outcomes, no significant differences were observed between groups. Potential effects might be better identified using qualitative methodology or self-reporting measures in a larger sample.

## Introduction

Parents of critically ill infants requiring care in a neonatal intensive care unit (NICU) often experience high levels of emotional stress, anxiety, and depressive symptoms (Grunberg et al., 2019; Harris et al., 2018; Petersson et al., 2025; Roque et al., 2017). They can experience continuing feelings of uncertainty and are at risk of developing posttraumatic stress after their infant is discharged from the NICU (Malin et al., 2022). Parental uncertainty combined with the negative emotional impact and paradoxical experience of having an infant in need of NICU care may disrupt parents’ individual processes of parental and family role attainment (Malin & Johnson, 2019), leading to decreased family functioning, including difficulties in verbal communication and problem-solving (Treyvaud et al., 2014).

Parents often provide important support to each other, but the NICU experience and associated individual mental health symptoms might negatively affect their future relationships, family functioning, and family wellbeing (Roque et al., 2017; Petersson et al., 2025). Therefore, Family-Centered Care is important for supporting parents in NICU with therapeutic relationships, clear communication, and acknowledging parents’ and families’ different needs (Adama et al., 2022; Kharrat et al., 2018; Krick et al., 2020). Moreover, parents suggested that medical providers should ask more open-ended questions, such as “What do you need?” and “What are your expectations for us?” to indicate genuine concern and family-centered care (Kodjebacheva et al., 2017). Parents identify nurses as key parts of this process since nurses accompany them more than other professionals during infants’ NICU care. Therefore, nurses must be interested in family processes and provide support according to parents’ expressed needs (DiBari & Rouse, 2023; San Rafael-Gutiérrez et al., 2020).

Moreover, studies have shown the need for targeted interventions to improve the wellbeing and functioning of parents and families after the birth of infants requiring NICU care (Adama et al., 2022; Galea et al., 2022; Gralton et al., 2022; Lundqvist et al., 2019). In line with this, there is a nurse-led intervention called the Family Assessment and Intervention Model developed in Canada, which focuses on the family as a system (Bell & Wright, 2015; Shajani & Snell, 2019). This intervention model has been adapted to Swedish culture in the form of the Family Health Conversation (FamHC) model (Benzein & Saveman, 2008) and has shown promising results. The core components of FamHC are narrating, listening, and reflecting on family experiences and challenges in finding new meaning in the situation (Benzein & Saveman, 2008; Östlund et al., 2015; Persson & Benzein, 2014). FamHC has been used and evaluated in different Swedish contexts, such as pediatric oncology (Marklund et al., 2018), adult intensive care (Hollman Frisman et al., 2018), and stroke care (Pusa et al., 2022; Sundin et al., 2016) with promising results for promoting family members’ individual wellbeing and functioning from a systemic family approach. In addition, an interview study of families with an adult family member in need of intensive care showed that FamHC contributed to families working together through their experiences, improved understandings of each other, strengthened togetherness within the family, and helped the family to move on (Hollman Frisman et al., 2018). The FamHC model has not yet been evaluated in families of infants receiving NICU care.

Today, in clinical practice, NICU care does not focus on the needs of the entire family or the family as a system. Therefore, it is important to investigate whether interventions with a systemic family focus, such as the FamHC model, might reduce the negative impacts of NICU infant care on individual and family health-related outcomes in this context.

Therefore, this study aimed to investigate the effects of the FamHC model on self-reported mental health, family wellbeing, and family functioning in parents of infants requiring mechanical respiratory support during NICU care.

## Methods

### Design

This prospective study used questionnaires administered three times to parents whose newborn infants required mechanical respiratory support during NICU care. This study was approved by the Regional Ethical Review Board in Linköping, Sweden (D-nr:2015/83–31, 2017/248–32).

### Procedure, Setting, and Participants

This study was conducted in Southern Sweden at three Level II NICUs: two units caring for newborns at 30 weeks of gestation and one unit at 27 weeks of gestation. The participants were recruited between June 2015 and March 2019. All units provided standard care based on the concepts of Family-Centered Care, Kangaroo Mother Care, and the Neonatal Individualized Developmental Care and Assessment Program. Therefore, the parents were invited to stay with their infants during the entire hospital admission and had the opportunity to perform neonatal home care before hospital discharge.

The inclusion criteria were cohabiting Swedish-speaking parents whose newborn infants required neonatal intensive care with mechanical respiratory support (i.e., a respirator or Continuous Positive Airway Pressure [CPAP]). Families were included regardless of the gestational age of infants and number of infants born (i.e., families with twins). In addition, families transferred to specialized intensive care units at a regional university hospital or another NICU were eligible for inclusion in the study.

Specified contact nurses at the three NICUs provided oral and written information to parents when their infants were in a more stable phase 1 to 6 weeks after birth. Consenting mothers and non-birthing parents (nb-parents; one woman and the rest men) were randomized to the FamHC intervention group or the control group. A priori power analysis was conducted with G* Power to estimate the minimum sample size required to identify a small effect of the intervention with 80% certainty and α set to .05. The analysis results determined that 90 parents were required for each of the intervention and control groups. The analysis was based on the intention to compare the group means for family functioning using an independent samples *t*-test.

### Intervention: The FamHC Model

The intervention was planned as a series of three conversations with 2- to 4-week intervals according to the FamHC model (Benzein et al., 2008), initiated 2 to 6 weeks after inclusion. Each conversation lasted approximately 1 hour and was led by two conversational leaders, following the family, through the entire FamHC series. All conversational leaders received training in using the FamHC model at the affiliated university and in using a checklist of the models’ core components (Östlund et al., 2015). Six conversational leaders conducted the conversations: five specialist nurses in pediatric and intensive care and one researcher in family systems nursing. The conversation series was implemented as a family intervention with both parents participating together. The parents chose the location for the conversations; the first took place in the NICU, while the following conversations, with a few exceptions, were held in the families’ own homes. The first conversation began with socialization. The parents then narrated their own experiences and listened to their partners’ experiences of having a child in need of NICU care. Conversational leaders asked open-ended circular questions to stimulate reflection. Such questions often begun with “how?” “where?” or “what?”; for example, “What are you most worried about right now?,” “What do you think when you hear your partner describe his or her worries?,” and “Which strengths and resources do you have together as parents?.” Circular questions extend the understanding and identification of relational patterns. They can facilitate parents to reflect on differences in feelings, thoughts, and behaviors and the importance of these differences for wellbeing within the family unit (Tomm, 1988). Parents talked about their own and the family’s situations and identified what was most important to talk about. The second conversation focused on the parents’ identified problems and their common resources in relation to the current situation. The third conversation focused on the future and process of change that the family had gone through. When the series of conversations was completed, a closing letter was sent 3 weeks after the last conversation. The closing letter summarized the conversations in a structured manner using the parents’ own words and was intended to be meaningful for them to read to keep the family conversation alive. Further, the letter was intended to confirm the family’s suffering and show that the conversational leaders had listened to the parents’ stories. The family’s strengths and resources were highlighted as the basis for parents’ further reflections (Moules, 2009). A total of 45 families participated in the FamHC. Most families participated in three (*n* = 18) or two (*n* = 17) conversations. Some families were satisfied with only one conversation (*n* = 5), while others wanted four conversations (*n* = 5).

### Data Collection

Data were collected using a study-specific questionnaire that included demographic and validated self-report measures for assessing mental health symptoms, family wellbeing, and family functioning. The parents in the intervention and control groups received the first questionnaire at 1–6 weeks after their infant’s birth, and at 5 and 8 months after inclusion in the study, respectively. Both groups responded to the same questionnaire on all three occasions. Two reminders were sent to participants who did not respond. A total of 147 parents (intervention group, *n* = 72; control group, *n* = 75) completed the first questionnaire. At the follow-up 5 months after inclusion, 113 parents (intervention, *n* = 62 vs. control, *n* = 51) responded, while 92 parents (intervention, *n* = 48 vs. control, *n* = 44) responded at 8 months. The infants were born at a gestational age of 22–40 weeks.

### Measures

#### Hospital Anxiety and Depression Scale

The Hospital Anxiety and Depression Scale (HADS) measures self-reported anxiety and depressive symptoms using two subscales, with higher scores indicating higher symptom levels (Zigmond & Snaith, 1983). The proposed cutoff scores for the subscales were as follows: normal range (0–7), possible presence (8–10), and probable presence (11–21; Snaith, 2003). In this study, the subscale total scores and the cutoff ≥8 were used. The HADS has been widely used and validated in adults (Russell et al., 2021; Saboonchi et al., 2013). In this sample, Cronbach’s alpha was .87 for HADS anxiety and .85 for HADS depression at baseline. Additional details are presented in Table 1.

**Table 1.** Overview of Self-Reported Measures.

Measures	Constructs	Items	Scales	Score ranges	Cutoff values
HADS	Anxiety and depression	14 Two subscales		0–21	≥8
PCL-S	Posttraumatic stress	17 Total scale		17–85	≥50
F-SOC-S	Family coherence and wellbeing	12 Total scale		12–84	-
GFS	Family functioning	12 Total scale		12–48	≥2

HADS = Hospital Anxiety and Depression Scale; PCL-S = Posttraumatic Stress Disorder Checklist-Specific; F-SOC-S = Family Sense of Coherence Scale-Short Form; GFS = General Family Functioning Scale.

#### Posttraumatic Stress Disorder Checklist-Specific

The Posttraumatic Stress Disorder Checklist-Specific (PCL-S) is designed to measure the posttraumatic stress symptoms following a traumatic life event (Weathers, 1993). The PCL-S consists of a self-report rating scale comprising 17 key symptoms (items), with higher scores indicating higher symptom levels. The PCL-S has been validated and used in several languages (Yao et al., 2003). Further, it has been used to follow-up with parents after discharge from the NICU (Schecter et al., 2020). Cronbach’s alpha for the sample was .91 at baseline. Additional details are presented in Table 1.

#### Family Sense of Coherence Scale-Short Form

The Family Sense of Coherence Scale-Short Form (F-SOC-S) was designed to measure a family’s sense of coherence and is linked to family wellbeing and quality of life. The instrument is based on three dimensions: the family perceives the environment as comprehensible, manageable, and meaningful (Antonovsky & Sourani, 1988). The instrument comprises 12 items, with higher scores indicating a stronger perception of family coherence. The instrument has shown good measurement properties in Swedish palliative care (Benzein & Berg, 2003) and has been validated among Chinese childbearing parents and with healthy newborns (Ngai & Ngu, 2014, 2016). Cronbach’s alpha for the sample was .84 at baseline. Additional details are presented in Table 1.

#### General Family Functioning Scale

The General Family Functioning Scale (GFS) is a self-report scale that measures family functioning which is defined as a process based on verbal communication and problem-solving among family members over time (Epstein et al., 1983). The GFS total score is based on the mean value of all 12 items, with lower scores indicating better family functioning. A score of two or higher indicated impaired family functioning. This scale is widely used for the follow-up of family functioning in this context (Mussatto et al., 2021; Pinelli, 2000; Treyvaud et al., 2014). Cronbach’s alpha for the sample was .87 at baseline. Additional details are presented in Table 1.

### Statistical Analyses

Descriptive statistics were used to present the parents’ characteristics in the intervention and control groups as absolute frequencies and percentages, mean values (*M*), and standard deviations (*SD*). Potential differences between the intervention and control groups were explored using the Mann–Whitney U test, Pearson’s chi-square test, and Fisher’s exact test. To evaluate the effects of the intervention, the total score of each instrument was modeled using linear mixed-effects models. Based on theoretical discussion and consensus in the research group, the analyses were adjusted for gender, age, and whether the participant had any prior children using fixed effects. The analyses also accounted for the dependency of observations from members of the same family by including family as a random effect. Analyses were conducted using IBM SPSS Statistics for Windows (version 27.0; IBM Corp, Armonk, NY) and the lme4 package (Bates et al., 2015) in the R statistical environment (R Core Team, 2025).

## Results

### Participant Characteristics

A total of 147 parents responded to the questionnaire at baseline. Their mean age was 32.3 years, and the majority were born in Sweden. All parents lived with their partners, and almost all perceived their financial status as good or very good. Approximately half of the parents had a university degree, and the majority were working full time. A few parents had previously received pharmacological treatment for anxiety or depression, whereas more than half had received support from a counselor or psychologist in conjunction with the NICU admission. No statistically significant differences were identified between the intervention and control groups, except for occupation (*p* = .019). Additional details are presented in Table 2.

**Table 2.** Baseline Characteristics—Intervention and Control Group.

Variabels	All (*n* = 147)	Intervention (*n* = 72)	Control (*n* = 75)
Age, mean (*SD*) [min–max]	32.3 (5.2) [22–52]	31.7 (5.1) [24–52]	32.8 (5.2) [22–47]
Country of birth, *n* (%)			
Sweden	141 (95.9)	70 (97.2)	71 (94.7)
Other	6 (4.1)	2 (2.8)	4 (5.3)
Cohabitation, *n* (%)			
Living alone	-	-	-
Cohabitating	147 (100.0)	72 (100.0)	75 (100.0)
Previous children, *n* (%)			
Yes	50 (34.0)	21 (29.2)	29 (38.7)
No	97 (66.0)	51 (70.8)	46 (61.3)
Education, *n* (%)			
Lower than secondary school	2 (1.4)	1 (1.4)	1 (1.3)
Secondary school	7 (4.8)	4 (5.6)	3 (4.0)
Upper secondary school	63 (42.9)	33 (45.8)	30 (40.0)
University	75 (51)	34 (47.2)	41 (54.7)
Occupation, *n* (%)			
Working full time	112 (76.2)	50 (69.4)	62 (82.7)
Working part time	16 (10.9)	12 (16.7)	4 (5.3)
Student	8 (5.4)	5 (6.9)	3 (4.0)
Other	11 (7.5)	5 (6.9)	5 (6.7)
Economics, *n* (%)			
Very good	38 (25.9)	17 (23.6)	21 (28.0)
Good	105 (71.4)	53 (73.6)	52 (69.3)
Poor	3 (2.0)	1 (1.4)	2 (2.7)
Very poor	1 (0.7)	1 (1.4)	-
Medical treatment for emotional distress, *n* (%)			
Yes	8 (5.4)	4 (5.6)	4 (5.3)
No	139 (94.6)	68 (94.4)	71 (94.7)
Conversational contact with counselor/psychologist, *n* (%)			
Yes	80 (54.4)	43 (59.7)	37 (49.3)
No	67 (45.6)	29 (40.3)	38 (50.7)

### Self-Reported Mental Health

More than half of the participants scored normal values for HADS Anxiety at baseline. Furthermore, the scores revealed an overall trend of decreasing anxiety symptoms over time in both groups (Tables 3 and 4).

**Table 3.** Self-Reported Mental Health and Family Functioning in the Intervention (n = 72) and Control (n = 75) Group.

Variables (mean, *SD*)	Baseline		5 months		8 months	
	Intervention	Control	Intervention	Control	Intervention	Control
*Self-reported mental health*						
HADS Anxiety	7.3 (4.2)	6.9 (4.7)	5.2 (3.8)	5.9 (4.5)	4.9 (3.1)	4.7 (3.5)
HADS Depression	4.1 (3.8)	4.0 (3.4)	3.0 (3.3)	3.8 (3.0)	2.6 (2.4)	3.5 (2.8)
PCL-S	29.0 (10.8)	28.9 (10.7)	25.5 (11.2)	26.4 (8.4)	23.3 (7.1)	24.9 (9.4)
*Self-reported family functioning*						
F-SOC-S	53.5 (5.9)	55.6 (5.0)	53.7 (6.6)	53.8 (5.3)	53.8 (5.6)	55.2 (5.9)
GFS	1.4 (0.4)	1.4 (0.3)	1.4 (0.4)	1.4 (0.4)	1.3 (0.4)	1.5 (0.5)

HADS = Hospital Anxiety and Depression Scale, PCL-S = Posttraumatic Stress Disorder Symptom Checklist-Specific, GFS = General Family Functioning Scale, F-SOC-S = Family Sense of Coherence Scale—Short Form.

**Table 4.** The Proportion of Participants With Symptoms of Mental Health Issues and Poor Family Functioning at Baseline (T1), at 5 Months (T2), and at 8 Months After Inclusion.

Outcome variable (normal range)	HADS anxiety (<8)			HADS depression (<8)			PCL-S (<50)			GFS (<2)		
	T1	T2	T3	T1	T2	T3	T1	T2	T3	T1	T2	T3
Not within normal range (all), *n* (%)	60 (41.4)	30 (26.8)	17 (18.5)	18 (12.2)	10 (8.9)	7 (7.6)	6 (4.8)	2 (1.9)	0	11 (7.5)	12 (10.7)	9 (9.8)
Intervention group	32 (44.4)	14 (22.6)	11 (22.9)	10 (13.9)	5 (8.1)	4 (8.3)	4 (6.6)	2 (3.6)	0	7 (9.7)	9 (14.5)	4 (6.2)
Control group	28 (38.4)	16 (32.0)	6 (13.6)	8 (10.7)	5 (10.0)	3 (6.8)	2 (3.1)	0	0	4 (5.3)	3 (6.0)	5 (11.4)

HADS = Hospital Anxiety and Depression Scale; PCL-S = Posttraumatic Stress Disorder Symptom Checklist-Specific; GFS = General Family Functioning Scale.

The vast majority scored normal values for HADS Depression at baseline. There was an overall decreasing trend in depressive symptoms over time, except between 5 and 8 months in the intervention group (Tables 3 and 4).

Overall, a few parents reported symptoms of post-traumatic stress disorder (PTSD) according to the PCL-S at baseline. In addition, there was an overall trend of decreasing PTSD symptoms over time in both groups (Tables 3 and 4).

### Family Wellbeing

The mean values of family wellbeing according to the F-SOC-S total scores were stable over time (Table 3).

### Family Functioning

Overall, the vast majority of parents reported normal family functioning at baseline according to the GFS. In addition, the proportion varied longitudinally in both groups. At 8 months, 93.8% in the intervention group reported normal family functioning compared to 88.6% in the control group. The mean values for the GFS were longitudinally stable (Tables 3 and 4).

### Estimated Effects of the Intervention

The effects of the intervention (receiving FamHC) were estimated by comparing the outcomes of the intervention and control groups, adjusting for gender, age, family, occupation, and whether the participant had any prior children.

Symptoms of anxiety (HADS-A): The estimated effect of the intervention was 0.30 points lower on the HADS Anxiety subscale after 5 months, but 0.63 points higher after 8 months.

Symptoms of depression (HADS-D): The estimated effect of the intervention was 0.33 points lower on the HADS Depression subscale 5 months after inclusion in the study and 0.04 points lower after 8 months.

PTSD symptoms (PCL-S): The estimated effect of the intervention was 2.36 points lower on the PCL-S scale 5 months after inclusion in the study and 1.33 points lower after 8 months.

Family wellbeing (F-SOC-S): The estimated effect of the intervention was 2.43 points higher on the F-SOC-S scale 5 months after inclusion in the study and 0.27 points higher after 8 months.

Family functioning (GFS): The estimated effect of the intervention was 0.08 points higher on the GFS scale 5 months after inclusion in the study and 0.14 points higher after 8 months.

Overall, all evaluated outcomes revealed considerable variation, and the estimated changes were not statistically significant at the 0.05 level (Table 5).

**Table 5.** Estimated Effects of the Intervention (Family Health Conversations).

Measures	Follow-up	n	Estimated effect^a^	95% CI	*p*-Value
HADS-A					
	5 months	103	−0.30	−1.86, 1.25	0.71
	8 months	86	0.63	−1.13, 2.37	0.50
HADS-D					
	5 months	104	−0.33	−1.39, 0.73	0.56
	8 months	87	0.04	−1.12, 1.18	0.95
PCL-S					
	5 months	86	−2.36	−6.11, 1.38	0.24
	8 months	67	−1.33	−5.99, 3.25	0.58
GFS					
	5 months	104	0.08	−0.07, 0.23	0.31
	8 months	87	0.14	−0.01, 0.28	0.07
F-SOC-S					
	5 months	90	2.43	−0.02, 4.87	0.06
	8 months	73	−0.27	−3.18, 2.65	0.86

HADS = Hospital Anxiety and Depression Scale, PCL-S = Posttraumatic Stress Disorder Symptom Checklist-Specific, GFS = General Family Functioning Scale, F-SOC-S = Family Sense of Coherence Scale–Short Form.

a Adjusted for gender, age, family, full-time employment, and whether the participant had any prior children.

## Discussion

The results showed that the FamHC intervention had no statistically significant effects on parents’ mental health symptoms, family wellbeing, or family functioning. Regardless of the intervention, mental health symptoms, such as anxiety, depression, and PTSD, decreased over time, while family wellbeing and functioning were stable.

Although the results indicated positive intervention estimates for all outcomes, no significant effects of FamHC were identified. The sample size in the present study might have been too small to evaluate the interventional effects. A previous Danish multicenter study with a sample of 468 patients and 322 family members (*n* = 322) evaluated FamHC in adult patients with heart failure and their families. Compared to the control group, intervened patients and family members perceived more knowledge about the illness, reported improvements in feedback within the family and decision-making capability, and better interactions with the nurses (Østergaard et al., 2021). These results are somewhat contrary to our findings and might be due to differences in sample size and choice of outcome measures.

The non-significant findings may also be due to insufficient intervention delivery, that is, not all of the families received the recommended three conversations, rather than the intervention’s lack of impact. Parents’ mental health issues, family wellbeing, and family functioning might have improved by natural recovery or just because time has passed, and the participants in both study groups might have had the opportunity to discuss their feelings within the family and with friends. This have been found to constitute important support for many parents and might have improved their mental health, family wellbeing, and family functioning (Flacking et al., 2019; Persson et al., 2024) consequently decreasing the effects of the FamHC intervention. In addition, in the present study, more than half of the parents received professional support, for example, from a counselor or a psychologist, which might have decreased the effects of FamHC.

Previous research indicated that family wellbeing and functioning are affected after receiving NICU care (Malin & Johnson, 2019; Treyvaud et al., 2014). In a U.S. study, the prevalence of family dysfunction after NICU care was considerably higher than in our study (Mussatto et al., 2021). In the present study, family wellbeing and functioning could be considered good and were longitudinally stable; the parents and families generally felt well, were living together, and reported having a good financial situation. Therefore, families with the greatest need for support were not included in our study. This may have affected the results and generalizability of the findings to other populations. Furthermore, a higher proportion of parents in the control group were working full time than in the intervention group. This difference might have contributed to the results. However, we controlled for occupation when analyzing the intervention effects to take this difference into account.

Moreover, the instruments’ potential lack of sensitivity may have affected the ability to identify the effects of FamHC. It is possible that the family wellbeing and family functioning scales do not capture change over time. Previous studies have discussed issues related to the psychometric properties (e.g., a potential lack of longitudinal sensitivity) for the measures applied to family wellbeing (Hochwälder, 2019) and family functioning (Bylund et al., 2016). In addition, another previous study reported the HADS displayed a lack of sensitivity and specificity (Pettersson et al., 2015). There is also a significant variation in the use of cutoff scores for the PCL-S in relation to NICU experience (McKeown et al., 2023). In this study, we used the cutoff ≥50. In a study by Schecter et al. (2020), ≥30 was used resulting in a higher prevalence. The most relevant cutoff for the present population should be further investigated.

FamHC interventions can be valuable even if they do not lead to reduced mental health symptoms, and self-reporting scales may not be the most effective evaluation method for the FamHC model. The potential effects may be better evaluated using other methods. A previous study by our research group evaluated the intervention using qualitative analysis based on interviews with parents receiving FamHC. These results reveal positive effects on family wellbeing and functioning. The FamHC was considered an opportunity for parents to co-create a comprehensive picture of what happened after their child was born. Parents felt validated and strengthened as individuals and families. They found that conversations supported them in processing experiences, improving their wellbeing, and being better equipped for the future (Åberg Petersson et al., 2021). Another Swedish qualitative study evaluated the effects of FamHC in families with critically ill adult family members in need of intensive care. The results showed that the intervention helped families better understand each other and become aware of their own family functioning (Ahlberg et al., 2020).

### Methodological Considerations

This study has several limitations. The sample size may have been too small to identify potential statistically significant effects of the intervention. The estimated number of participants needed, based on the power calculation, was not reached because of dropouts. Therefore, the results of this study could be enhanced if it was replicated with a larger sample. Further, not all families in the FamHC intervention group received the full intervention with at least three conversational meetings. The random allocation of parents within a NICU increases the risk of contaminating the intervention. However, the FamHC intervention is unlikely to have a considerable impact on standard care since implementing the model requires professionals to receive education and engage in clinical practice. In addition, most of the FamHC intervention was conducted by the research group and not by clinicians.

Participants who could speak and read Swedish and lived with a partner were included. In addition, most participants perceived their financial situation as good. Therefore, the final sample was based on parents who lacked cultural, social, and economic diversity, which may have affected the results and limited their generalizability. Further studies should aim to include families with socioeconomic challenges, since they probably need more support, such as that provided by the FamHC intervention. In addition, we did not collect information about the infants’ diagnosis or length of care, which would have enriched the analysis and may have affected the results.

Owing to the lack of previous studies, the choice of adjusting variables for evaluating the effects of FamHC was based on a theoretical discussion and consensus within the research group. This could be considered a limitation because other variables might also have been appropriate. However, the choice of adjusted variables was limited by sample size.

## Conclusions

Regardless of the intervention, mental health symptoms, such as anxiety, depression, and PTSD, decreased over time, while family wellbeing and functioning were stable.

Although the results indicated positive estimates of the intervention for all outcomes, no statistically significant effects of FamHC were observed. The sample size in the present study might have been too small to evaluate the intervention, and the potential effects might have been better identified using a qualitative methodology.
